# Vaccine hesitation: attitudes of Turkish health worker parents towards adult and childhood immunisation

**DOI:** 10.1186/s12889-025-24100-5

**Published:** 2025-08-20

**Authors:** Emre Çelik, Çağrı Emin Şahin, Mehmet Sait Değer, Zeynep Meva Altaş

**Affiliations:** 1 Department of Pediatrics, Bursa City Training and Research Hospital, Bursa, Turkey; 2 Epidemiology Program, Institute of Health Sciences, Istanbul Medipol University, Istanbul, 34810 Turkey; 3 Department of Public Health, Cerrahpaşa Faculty of Medicine, İstanbul University- Cerrahpaşa, İstanbul, Turkey; 4 Department of Public Health, Faculty of Medicine, Marmara University, İstanbul, Turkey; 5 Department of Public Health, Health Sciences University- Gülhane Faculty of Medicine, Ankara, Turkey

**Keywords:** Healthcare professionals, Turkiye, Vaccine hesitancy, Vaccine refusal

## Abstract

**Background:**

This study aimed to evaluate the perceptions, attitudes, and beliefs of healthcare professionals who are also parents toward childhood vaccinations, as well as the sociodemographic and occupational factors associated with these attitudes.

**Methods:**

This cross-sectional research study employed a population of healthcare professionals who are also parents. Sociodemographic data, parental attitudes toward childhood vaccines (PACV Scale), and psychological vaccination antecedents (5 C Scale) were obtained through a Google Forms survey. Vaccine hesitancy was considered to be indicated by a PACV score of 50 or above. The five subdimensions of the 5 C scale are confidence, complacency, constraints, calculation, and collective responsibility. Elevated scores on a subdimension reflect more positive attitudes toward that dimension. A total of 374 individuals participated in this study.

**Results:**

The rate of delaying vaccinations due to reasons other than illness or allergy was 11.5%, while the rate of vaccine refusal was 8.0%. The survey revealed that 85.6% of respondents favored the administration of all vaccines on the Ministry of Health’s schedule to newborns. Based on the PACV scale, vaccine hesitancy was observed in 7.5% of participants (*n* = 28). A statistically significant negative association was found between PACV scores and higher levels of education and income (*p* < 0.05). A significant correlation was observed between PACV scores and a range of factors: gender, level of education, professional group, experience, income, and vaccine delay or rejection. The median PACV score for physicians was significantly lower than that of midwives, nurses, and public health officers (*p* < 0.001).

**Conclusions:**

Despite predominantly positive participant attitudes towards childhood vaccination, supplemental educational outreach programs focusing on low-income communities and parents who decline vaccination are recommended. Qualitative studies are necessary to elucidate the motivations and perspectives underlying vaccine hesitancy within the healthcare profession.

**Supplementary Information:**

The online version contains supplementary material available at 10.1186/s12889-025-24100-5.

## Background

Vaccine hesitancy, defined by the World Health Organisation (WHO) as the delay or refusal of vaccines, poses a significant threat to global health [1]. Healthcare professionals, who are considered the most trusted advisors and influencers of vaccination decisions, play an important role in this dynamic [2]. Indeed, the decisions of parents regarding immunisations for their children are influenced by their interactions with healthcare providers, and this interaction plays an important role in reducing or exacerbating parental hesitancy to vaccinate. Knowledge of how to overcome parental hesitancy about childhood vaccines and how to positively support parents’ attitudes towards childhood vaccines remains weak. Understanding these attitudes and the dynamics of parent/healthcare worker interactions is necessary for the development of effective communication strategies and interventions to improve immunisation rates [3].

At the same time, vaccine hesitancy among healthcare professionals leads to lower vaccine uptake among patients, decreased likelihood of recommending various vaccines to patients and lower self-efficacy and commitment to addressing patients’ vaccine hesitancy [4]. Yet, Research has shown that effective communication between healthcare professionals and parents can address concerns and misunderstandings about vaccines and lead to higher acceptance rates [5]. It has also been shown that poor communication with healthcare professionals and misinformation may lead to patients’ increased vaccine hesitancy and refusal [6]. However, many qualitative studies that examined healthcare workers have shown the presence of vaccine hesitancy among this population [7]. Studying parents’ vaccination attitudes among health workers is crucial to address vaccine hesitancy. Understanding healthcare professionals’ vaccine hesitancy is critical to understanding vaccination behavior.

Furthermore, considering this background, examining the immunisation attitudes of parents who are health workers can offer an important perspective in understanding vaccine hesitancy, both as the most trusted advisors and influencers of the immunisation decisions of the parents they serve and as decision-makers about their own children’s immunisations as parents.

In Turkiye, few studies have examined the vaccine hesitancy of healthcare professionals who are also parents, and no study has been found that examined their attitudes towards childhood vaccines. Despite their critical role in ensuring public confidence in vaccines, healthcare workers who are parents are also exposed to situations and factors that determine their attitudes towards childhood vaccines and vaccine hesitancy. Therefore, this study aimed to evaluate the beliefs and the sociodemographic and occupational factors associated with healthcare professionals who are parents in relation to their perceptions and attitudes towards childhood immunisations.

## Materials and methods

### Type of research and study population

The design of this research was a cross-sectional study. There are approximately 1 million healthcare professionals in Turkey. The population of the study consisted of healthcare workers who have children. Because the exact number of people in Turkey who work in health services and have children is not known, this study aimed to include as many participants as possible by applying the convenience sampling method. For the sample size of the study, type I error 0.05, type II error 0.20 and power 0.80 were accepted and the minimum sample size was calculated as 384 people. The link to the Google form prepared for the questionnaire was shared with healthcare professionals in 12 healthcare facilities in different provinces of Turkey where the researchers had previously worked and are currently working, healthcare professionals known to the researchers individually, and social media groups consisting of healthcare professionals in which the researchers are involved through channels such as email and WhatsApp. In this way, healthcare professionals in different regions and healthcare settings were invited to participate in the study. Thus, the research was completed with 385 people through a web-based survey application. Eleven incomplete and incorrect forms were excluded from the final evaluation, resulting in data for 374 people being included in the analysis. The research was conducted between March and July 2024.

### Measurement tools

The questionnaire was developed for the purposes of this study and consists of three sections. The participants were invited to complete the questionnaire, which was organised via Google Form Sociodemographic data (age, gender, education, occupation, title, professional experience, income, number of children, and children’s ages) were elicited in the first section using questions grounded in the relevant literature. The other two sections consisted of the Parent Attitudes about Childhood Vaccines (PACV) survey and The 5 C Psychological Antecedents of Vaccination measure, for which Turkish validity and reliability studies were conducted.

### The Parent Attitudes about Childhood Vaccines (PACV) survey

This survey consists of 15 items and 3 sub-scales (i.e. behaviour, attitude and safety-effectiveness) that evaluate parents’ attitudes and hesitation regarding childhood vaccinations. The scale was developed by Opel et al. in 2011, and the Turkish validity and reliability study of the scale was carried out by Mutlu in 2023 [8, 9]. Participants can rate each item in the survey according to a scale ranging from 0 to 2. A rating of 2 means the hesitant answer, a rating of 1 means ‘I don’t know’ or ‘I’m not sure’ and a rating of 0 points to the answer without hesitation. After assigning scores to each item, they are summed to calculate the total raw score, which ranges between 0 and 30. The total raw score is then converted into a 0-100 scale using a simple linear transformation method developed by *Opel et al.*, as outlined in their score conversion table. Participants whose total converted score is less than 50 do not have vaccine hesitancy, and participants with a score of 50 and above are said to have vaccine hesitancy. The scale’s reliability, indicated by Cronbach’s alpha, was 0.845 in the Turkish validation study and 0.842 in the original [8, 9]

### The 5C psychological antecedents of vaccination scale

This measure, which is a 5-point Likert-type scale, helps measure hesitation about vaccination. It consists of 15 items and 5 sub-scales (i.e. confidence, complacency, constraint, calculation and collective responsibility). Participants can answer each item with a score ranging from 1 (‘strongly disagree’) to 5 (‘strongly agree’). The confidence subscale indicates the trust in the efficacy and safety of vaccines, vaccination policies, and the reliability and competence of healthcare services and healthcare professionals. The complacency of the 5 C scale measures the attitude that the risks of vaccine-preventable diseases are low and vaccination is not seen as a necessary preventive action. The constraint sub-scales includes items related to the physical availability of a vaccine, its affordability and the individual’s willingness to pay for it, access to health services and the individual’s capacity to understand the need for vaccination (i.e. health literacy). The calculation sub-scales relates to individuals’ searches for comprehensive information on disease, vaccine risks and the need for vaccination. The collective responsibility sub-scales indicates to the willingness to contribute to herd immunity through individual vaccination. There are three questions in each sub-scales. The higher the score obtained in a sub-scales, the more apparent is that sub-scales’s attitude in the individual respondent. The scale was developed by Betsch et al. and the Turkish validity and reliability study was carried out by Demir et al. in [10, 11]. The scale, developed by Betsch et al. [10], exhibited a Cronbach’s alpha ranging from 0.73 to 0.80 for each subscale [10]. Subsequently, Demir et al. [11] conducted a Turkish validation and reliability study, reporting a Cronbach’s alpha of 0.78 [11].

### Statistical analysis

For the statistical analysis, the PACV survey and the 5 C scale were accepted as dependent variables. The Statistical Package for the Social Sciences (SPSS) software version 22.0 was used to conduct the statistical analysis. Continuous variables were expressed as mean ± standard deviation (SD) and median (min-max). Categorical variables were expressed as number and percentage (%). The Kolmogorov-Smirnov and Shapiro-Wilk tests were performed for the normality analysis of the data, and the skewness and kurtosis values of the scales with *p* < 0.05 were examined. The values with skewness and kurtosis values in the range of ± 2 were considered normally distributed, and parametric tests were applied. Nonparametric tests were used for values not falling within the range of ± 2.

Chi-square and Fisher’s exact tests were used to compare the categorical variables between groups. Student’s t test and a one-way ANOVA test were used for the statistical analysis of the normally distributed data, while the Mann-Whitney U test and the Kruskal-Wallis test were used to analyse the non-normally distributed data. Spearman’s rank correlation coefficient was performed to find the relationship between the continuous variables. In the statistical analyses, a p value of ˂ 0.05 was considered significant. Correlation analyses were performed between the models established by taking into account the parameters found to be significant in the analytical tests and the continuous variables, and logistic regression analysis was performed to predict the level of vaccine hesitancy according to the independent variables, model fits were evaluated, and the variables that contributed significantly to the model were examined.

### Ethics committee permission

For this study, ethics committee approval was obtained from the Istanbul Medipol University Ethics Committee on 15 February 2024 with protocol number 169. The people included in the study were informed about the research and asked to participate in the study. Our study was conducted according to the Declaration of Helsinki, and informed consent was obtained from all of the participants.

## Results

Responses from a total of 374 people were included in the study. The average age of the participants was 39.78 ± 7.30 years. The youngest participant was 26 years old, and the oldest participant was 73 years old. Of the 374 parent health workers who participated in the study, 11.5% stated that they delayed their children’s vaccinations for reasons other than illness or allergy, and 8% stated that they did not vaccinate their children. A large majority—85.6% of participants—reported wanting all of the vaccines in the vaccination schedule of the Turkish Ministry of Health to be administered when a new baby was born. Data on the sociodemographic characteristics and vaccination attitudes of the participants are shown in Table 1.

**Table 1:** Sociodemographic characteristics and vaccination attitudes of participants

Variable	Number (*n*)	Percentage (%)
Gender		
Male	169	45.2
Female	205	54.8
Education level		
Doctorate or higher	115	30.7
Bachelor’s degree	175	46.8
Master’s degree	72	19.3
High school	12	3.2
Profession		
Physician	181	48.4
Midwife/Nurse/Public health officer	120	32.1
Technician/technologist	17	4.5
Clerk	11	2.9
Paramedic/Emergency medical technician	6	1.6
Child development Specialist/Dietitian/Psychologist/Physiotherapist/Social worker	8	2.1
Occupational groups		
Physician	181	48.4
Midwife/Nurse/Public health officer	120	32.1
Pharmacist	31	8.3
Other	42	11.2
Specialty groups among physicians		
Internal medicine	75	20.1
General practitioner	54	14.4
General surgery	37	9.9
Basic medical sciences	15	4.0
Years of professional experience		
Less than 1 year	5	1.3
1–5 years	29	7.8
6–10 years	72	19.3
11–15 years	99	26.5
More than 15 years	169	45.2
Income Level		
Less than 35.000 TRY	8	2.1
35.000–50.000 TRY	139	37.2
50.000–90.000 TRY	107	28.6
More than 90.000 TRY	120	32.1
Age of youngest child		
0–2 years	84	22.5
2–5 years	105	28.1
5–15 years	144	38.5
Over 15 years	41	11.0
Have you ever delayed any of your child’s vaccinations (excluding illness or allergy)?		
I don’t know	8	2.1
Yes	43	11.5
No	323	86.4
Have you ever decided not to vaccinate your child (excluding illness or allergy)?		
I don’t know	5	1.3
Yes	30	8.0
No	339	90.6
If a new baby was born today, would you want all vaccinations in the ministry of health’s schedule to be administered?		
I don’t know	27	7.2
Yes	320	85.6
No	27	7.2

TRY: Turkish Lira

In our study, the Cronbach’s alpha coefficient of the PACV scale was 0.884. A total of 13.6% of the participants thought that children are vaccinated more than what is necessary. The percentage of participants who had no hesitancy about childhood vaccines was 81.8%. A total of 66.6% of the participants indicated that they trust the information they receive about vaccines, while 64.2% said that they can openly discuss their concerns about vaccines with their doctors. The percentage of those who were hesitant about childhood vaccines was 12.8%. Detailed information about the healthcare workers’ attitudes towards childhood vaccines is given in Table 2.

**Table 2:** Responses to the parent attitudes about childhood vaccines (PACV) scale

Question	Number (*n*)	Percentage (%)
How sure are you that following the vaccination schedule recommended by the ministry of health is a good idea for your child?		
No hesitation	295	78.9
Unsure	47	12.6
Hesitation	32	8.6
How much do you trust your child’s doctor?		
No hesitation	287	76.7
Unsure	53	14.2
Hesitation	34	9.1
Children receive more vaccines than they need.		
No hesitation	265	70.9
Unsure	58	15.5
Hesitation	51	13.6
I believe most of the diseases that vaccines prevent are serious.		
No hesitation	296	79.1
Unsure	18	4.8
Hesitation	60	16.0
It is better for my child to build immunity by getting sick than by getting vaccinated.		
No hesitation	47	12.6
Unsure	57	15.2
Hesitation	270	72.2
It is better for children to receive fewer vaccines at the same time.		
No hesitation	135	36.1
Unsure	89	23.8
Hesitation	150	40.1
I worry that my child could experience serious side effects from any vaccine.		
No hesitation	152	40.6
Unsure	72	19.3
Hesitation	150	40.1
I am concerned that some childhood vaccines may not be safe.		
No hesitation	251	67.1
Unsure	59	15.8
Hesitation	64	17.1
I worry that a vaccine may not prevent the disease it is supposed to.		
No hesitation	251	67.1
Unsure	54	14.4
Hesitation	69	18.4
Overall, I am hesitant about childhood vaccines		
No hesitation	306	81.8
Unsure	20	5.3
Hesitation	48	12.8
I trust the information I receive about vaccines.		
No hesitation	249	66.6
Unsure	59	15.8
Hesitation	66	17.6
I can openly discuss my vaccine concerns with my child’s doctor.		
No hesitation	240	64.2
Unsure	52	13.9
Hesitation	82	21.9

According to the results of the PACV survey, 7.5% (*n* = 28) of the participants had vaccine hesitancy (defined as a PACV score of 50 and above). A decreasing trend was observed in the PACV scores as the level of education and income level of the participants increased (*p* < 0.05). According to sociodemographic characteristics, the PACV score was significantly higher in women compared to men and in those with an income of 35,000 Turkish Liras (TRY) or less compared to the other participant income groups (*p* < 0.05), lower in those with doctoral education compared to the other participant education groups (*p* < 0.05) and lower in the physician group compared to the other participant occupational groups (*p* < 0.001). The median PACV score of the physicians who responded to the survey was significantly lower than the median score of the midwife/nurse/public health officer group [score:27] (*p* < 0.001). Detailed information is given in Table 3.

**Table 3:** Relationship between PACV scale, sociodemographic characteristics, and vaccination attitudes

Variable	PACV		PACV-A		PACV-B		PACV-C		
	Median	Min–Max	*P*	Median	Min–Max	*p*	Median	Min–Max	*p*
Gender*									
Male	20	0–63	0.025	0	0–4	0.19	2	0–10	0.62
Female	23	0–67		0	0–4		3.5	0–12	
Education level **									
High school^2^	26.5	13–53		0.5	0–3		3	0–9	0.37
Bachelor’s degree	23	0–67		0	0–4		3	0–10	
Master’s degree	20	7–67		0	0–4		2.5	0–12	
Doctorate or higher^1^	20	7–57	0.042	0	0–4	0	2	0–8	
Profession **									
Physician^1^	20	7–60	< 0.001	0	0–4	< 0.001	2	0–8	0.24
Midwife/Nurse/Public health officer^2^	27	0–67		0	0–4		4	0–10	
Pharmacist^2^	30	0–67		0	0–4		4	0–12	
Other^2^	28.5	13–60		1	0–4		4	0–10	
Specialty among physicians **									
Internal medicine	20	7–43	0.87	0	0–2	0.241	2	0–8	0.92
General practitioner	20	7–60		0	0–4		2	0–8	
General surgery	20	10–50		0	0–3		2	1–8	
Basic medical sciences	23	7–40		0	0–1		4	0–6	
Years of professional experience **									
< 1 Year	30	20–60	0.35	1	0–3	0.288	3	1–9	0.38

*PACV-A*, Behavior; *PACV-B*, General Attitude; *PACV-C*, Efficacy-Safety. *MWU, Mann-Whitney U; **KW, Kruskal-Wallis; ^1,2^: Indicates significant differences between groups

In terms of the 5 C scale scores, a significant difference was found in the complacency sub-scales according to the educational level of the participants (*p* = 0.001). The participants who had a doctorate level of education showed a lower level of complacency compared to the other participants. A significant difference was also found in the collective responsibility sub-scales of the 5 C scale (*p* < 0.001). The participants who had a doctorate had the highest collective responsibility scores. In addition, Physicians were found to have higher confidence in vaccines, health institutions and health professionals in subscale confidence of the psychological antecedents of immunisation scale, higher perception of risk posed by vaccine-preventable diseases in subscale constraints, and more willing to contribute to social immunisation in subscale collective responsibility compared to participants with other professions (*p* < 0.001, *p* < 0.001, *p* < 0.001). Likewise, significant differences were found in these sub-scales according to income level (*p* = 0.002 and *p* < 0.001). In the income group below 35.000 TRY, while vaccine complacency was found to be higher compared to other income groups, social responsibility was found to be lower. Those who decided not to vaccinate their children had a higher constraints score (*p* = 0.036), but no significant difference was observed in other subscales of the 5 C scale. The median value of the 5 C scale was significantly higher in those who delayed any vaccination of their children for reasons other than disease or allergy (*p* < 0.001). The median value was significantly higher in confidence and collective responsibility subscales (*p* < 0.001, *p* < 0.001), and the median value was significantly lower in constraints and complacency subscales (*p* < 0.001, *p* < 0.001) in those who supported the administration of all vaccines in the vaccination schedule of the Turkish Ministry of Health. The details of the 5 C scale scores are shown in Table 4. However, in our study, the Cronbach’s alpha coefficient of the 5 C Psychological Antecedents of Vaccination scale was 0.699.

**Table 4:** Relationship between 5C scale, sociodemographic characteristics, and vaccination attitudes

Variable	5 C		C1		C2		C3		C4		C5	
	Median	Min–Max	*p*	Median	Min–Max	*p*	Median	Min–Max	*p*	Median	Min–Max	*p*
Gender												
Male	4.11	2.24–5.13	0.414	5.6	1.87–7	0.411	2.33	1.4–4.67	0.723	2.8	1.4–5.6	0.992
Female	4.2	2.33–5.32		5.6	1.87–7		2.33	1.4–7		2.8	1.4–5.6	
Education level												
High school²	4.11	2.71–5.04		5.37	3.73–7		2.57	1.4–5.6		2.57	1.4–5.13	
Bachelor’s degree²	4.11	2.33–5.32		5.6	1.87–7		2.8	1.4–7		2.8	1.4–5.6	
Master’s degree	4.2	2.61–5.13		5.6	2.8–7		2.33	1.4–4.2		2.33	1.4–5.13	
Doctorate or higher¹	4.2	2.24–5.13	0.515	5.6	1.87–7	< 0.001	2.33	1.4–4.67	0.001	2.8	1.4–5.6	0.766
Profession												
Physician¹	4.2	2.24–5.13	0.575	5.6	1.87–7	< 0.001	2.33	1.4–4.67	< 0.001	2.33	1.4–5.6	0.581
Midwife/Nurse/Public health officer²	4.15	2.33–5.32		5.6	1.87–7		2.8	1.4–7		2.8	1.4–5.6	
Pharmacist²	4.01	2.61–4.85		5.6	1.87–7		2.33	1.4–7		2.33	1.4–5.6	
Other²	4.2	2.33–4.85		5.13	2.8–6.53		2.8	1.4–7		2.8	1.4–5.13	
Years of professional experience												
< 1 Year	4.29	4.01–5.13	0.326	5.6	3.73–7	0.102	2.8	1.4–3.73	0.424	2.8	2.8–5.13	0.057

*C1*, Confidence, *C2*, Constraints, *C3*, Complacency, *C4*, Calculation, *C5*, Collective Responsibility. ¹^,^² indicate significant differences between groups

The income level variable had a significant effect on the PACV scores (*p* = 0.072). Individuals with an annual income of less than 35,000 TRY had PACV scores 0.059 times lower than the other income groups (confidence interval (CI): 0.003–1.108). Individuals with an income between 50,000 and 90,000 TRY had PACV scores 0.059 times lower than the other income groups (CI: 0.0–0.252). The variable of wanting a baby to receive all vaccines on the vaccination schedule of the Ministry of Health also had a significant effect on the PACV scores (*p* < 0,01). The regression analysis of the PACV scores and related factors is shown in Table 5.

**Table 5:** Regression analysis of PACV scale scores and associated factors

	B	S.E.	Wald	Df	Sig.	Exp(B)	95% C.I.for EXP(B)	
							Lower	Upper
Gender (Female)	−0.95	0.76	1.53	1	0.21	0.38	0.08	1.73
Education level (High School)			1.01	3	0.79			
Education level (Bachelor’s Degree)	1.08	1.86	0.33	1	0.56	2.95	0.07	113.48
Education level (Master’s Degree)	0.17	1.92	0.01	1	0.92	1.19	0.02	51.41
Education level (Doctorate or Higher)	0.71	2.31	0.09	1	0.75	2.05	0.02	190.17
Profession (Other)			1.79	3	0.61			
Profession (Pharmacist)	1.36	1.45	0.8	1	0.34	3.92	0.22	68.48
Profession (Midwife/Nurse/Public Health Officer)	1.79	1.34	1.79	1	0.18	6.03	0.43	83.42
Profession (Physician)	0.96	1.39	0.47	1	0.49	2.61	0.17	39.88
Income (< 35.000 TRY)			6.99	3	0.07			
Income (35.000–50.000 TRY)	−2.82	1.49	3.57	1	0.05	0.05	0.01	1.10
Income (50.000–90.000 TRY)	−3.21	1.55	4.2	1	0.03	0.04	0.01	0.84
Income (> 90.000 TRY)	−5.36	2.03	6.95	1	0.00	0.01	0	0.25
Vaccination delayers	−0.97	1.10	0.78	1	0.37	0.37	0.04	3.29
Vaccination refusers	−1.82	0.93	3.80	1	0.05	0.16	0.02	1.00
All vaccines administered (Schedule)	3.79	0.76	24.4	1	0	44.52	9.87	200.65
Constant	−0.36	2.67	0.01	1	0.89	0.69		

*TRY* Turkish Lira

*Hosmer&Lemeshow = 3.92 (*p* = 0.864), **Nagelkerke r²= 0.603

## Discussion

The reasons why individuals and health professionals delay or refuse vaccinations can be varied and complex. These reasons include concerns about the content and safety of vaccines, doubts about the necessity of vaccines, personal beliefs or opinions, distrust of the health system, misinformation or incomplete information, individual experiences, fears about the side effects or long-term effects of vaccines, the belief that vaccines are made for commercial interests, belief in natural immunity or scepticism about scientific evidence of the benefit of vaccines [12, 13]. In studies involving 315 parents applying to a health centre in Vietnam and 191 participants with children aged 0–36 months in Germany, the rate of those who postponed vaccination of their children for reasons other than disease or allergy was 31.4%, and the rate of those who decided not to vaccinate was 13.7%−16.2% [14, 15]. A further study, conducted with 941 parents in Turkey, revealed that the rate of those who delayed vaccination for reasons other than disease or allergy was 3.7%, with the rate of those who did not vaccinate being 2.2%. In the present study, although the rate of parents who delayed vaccination for reasons other than illness or allergy (11.5%) and those who did not vaccinate their children (8.0%) was lower than that reported in the literature, the percentages were nevertheless high in terms of the danger of social immunisation. This may be due to the participants’ low risk perception of vaccine-preventable diseases. The fact that the participants in our study were healthcare professionals and considered themselves more knowledgeable about health-related issues than the general public may have contributed to their comfort level in vaccinating their children.

The attitudes and approaches of individuals towards vaccination are influenced by their perception of health, perception of disease and trust in healthcare professionals [16–19]. A study of the literature revealed that 73% of the participants trusted their child’s doctor [20]. In contrast, the present study revealed a higher rate of trust in child healthcare professionals (76.7%), though it should be noted that 20% of the participants expressed uncertainty or scepticism regarding the severity of preventable diseases caused by vaccines. This could be indicative of a low perception of disease risk among the participants. A further study involving 2,428 individuals in Turkey revealed that individuals prioritised vaccination for the purpose of safeguarding their own health and that of their relatives. The study also demonstrated that the provision of accurate and sufficient information regarding recommended vaccines had a favourable impact on vaccination status [21]. An additional study in the literature reported that 80% of the participants attributed the positive influence of primary healthcare providers on parents’ vaccination decisions to the providers [22]. Furthermore, it was demonstrated that vaccinated healthcare professionals were more inclined to recommend vaccination to their patients compared to unvaccinated healthcare professionals [23]. These results signify that healthcare workers significantly contribute to positive vaccination attitudes and behaviours. However, in order to address those with negative attitudes towards vaccination, studies that provide information and help to build confidence in vaccines are also necessary. In order to increase trust in healthcare professionals, it is important to understand the underlying causes of mistrust. In addition, there is a need to examine vaccine hesitancy and its causes among healthcare professionals. Qualitative studies on this subject are of critical importance.

Vaccine hesitancy and refusal are issues of mounting importance. Research conducted in a paediatric hospital in Ireland and in Vietnam revealed that the prevalence of parental vaccine hesitancy was 14.4% and 8.9%, respectively [24]. A further study in Turkey reported a figure of 7.6% for vaccine hesitancy among parents with children aged between 0 and 59 months [22]. In two recent studies conducted in Turkey, the percentages of vaccine hesitancy in parents of preschool-age children were reported as 13% and 17.2%, respectively [9, 25]. Torun et al. revealed that the pandemic influenza A/H1N1 vaccination rate was low (21.1%) among children whose parents were healthcare workers [26]. In a study conducted by De Araújo et al. with 453 healthcare workers, 27.8% had not received the recommended three doses of the hepatitis B vaccine, 40.4% had not completed the recommended triple viral vaccine (measles, mumps and rubella) vaccination programme for healthcare workers and 39.0% had not completed the vaccination programme for diphtheria and tetanus [27]. An international review by Verger et al. of healthcare workers who were also parents reported that their perceptions and attitudes towards childhood immunisations differed from each other [4]. In addition, it has been shown that the perceptions and knowledge of healthcare workers about the benefits and safety of vaccines are related to their own vaccination behaviours in terms of both allowing their own children to be vaccinated in accordance with a national vaccination programme and accepting the vaccines required for themselves [28]. In a study conducted by Öncel et al. among healthcare professionals, it was reported that 31.9% of the participants were hesitant about having their children vaccinated against COVID-19. In addition, 15.6% of those who responded to the relevant question reported that they would never approve the vaccination of their children, even if the safety and efficacy of the COVID-19 vaccine in children were more robustly demonstrated in the future [29]. In contrast, the present study documented a comparatively lower rate of vaccine hesitancy, with a figure of 7.5%, a finding that is in alignment with the extant literature. This discrepancy may be attributed to the fact that our study was conducted with healthcare workers who were also parents. Furthermore, cultural and societal influences may play a significant role in shaping vaccine hesitancy, underscoring the need for nuanced approaches to healthcare promotion in diverse global contexts. Notwithstanding, healthcare workers are likely to possess a greater degree of evidence-based knowledge regarding vaccines in comparison to the general population, which is likely to result in a lower prevalence of vaccine hesitancy among healthcare workers.

The population’s perceptions of the risk of contracting a vaccine-preventable disease, as well as the difficulties they face in accessing vaccines, may result in vaccine hesitancy. Conversely, confidence in vaccines and a desire to protect society through vaccination have been identified as factors that can mitigate vaccine hesitancy [30, 31]. A review of the extant literature reveals that the most commonly cited reason for vaccine hesitancy in countries is concerns about vaccine safety and side effects [24]. In a particular study, misgivings regarding the safety of the influenza vaccine for the foetus were identified as a hindrance to the vaccination of expectant mothers against influenza [32]. In the present study, the characteristics of the participants, such as confidence in vaccination, high collective responsibility and willingness to be vaccinated, were found to be similar to those reported in the literature. The implementation of policies aimed at enhancing the health literacy level of the population and instilling confidence in health institutions with regard to vaccination has the potential to mitigate vaccine hesitancy.

A multitude of sociodemographic characteristics have been demonstrated to exert an influence on both the propensity to vaccinate and the reluctance to do so. Such characteristics encompass age, gender, educational attainment, occupation, income level, and religious beliefs. A plethora of studies have demonstrated that factors such as young age, low education and income level, and religious beliefs are associated with vaccine hesitancy [33–36]. In other studies, it was demonstrated that vaccine hesitancy in women was higher than in men [37, 38]. The present study demonstrated a strong correlation between adherence to the Ministry of Health’s vaccination schedule and positive attitudes towards vaccination. The study also revealed that vaccine hesitancy was more pronounced among women, individuals with low income and education levels, and non-physician healthcare workers. The finding that physicians and healthcare workers consider themselves more knowledgeable about health-related issues and/or work under conditions of extreme fatigue may have led them to be more comfortable with delaying vaccination. Furthermore, gender roles and the primary role of mothers in childcare may lead to a greater assumption of responsibility for vaccination decisions and increased anxiety regarding vaccines. Furthermore, individuals with lower levels of education and income may have reduced awareness and health literacy, which could be associated with higher levels of vaccine hesitancy.

### Limitations and strengths

Because this study was conducted through a questionnaire, the findings obtained are based on the statements of health workers. This may have caused social desirability bias. Also, because the occupational group of nurses and physicians constituted the majority of the participants in the study, the opinions of other types of healthcare professionals may not have been adequately represented. This imbalance constitutes a limitation based on the participants’ demographics in terms of generalising the results of the study to all healthcare professionals.

In addition to its limitations, the study also has strengths. This study conducted with healthcare workers focused on a current and global public health problem. According to our research, there is no study in the literature that evaluated vaccine hesitancy and related factors among healthcare workers who are also parents. Because healthcare professionals are role models who can influence the health behaviours of other members of society, understanding their vaccine hesitancy and its underlying causes will be useful in guiding vaccination services and reducing vaccine refusals. The different types of healthcare professionals could not be included in similar numbers. but nevertheless there was at least some diversity and this represented a strength of the study.

## Conclusion

It is imperative to comprehend the factors that precipitate vaccine hesitancy among healthcare professionals, as this knowledge is instrumental in the prevention of such hesitancy within the community and the enhancement of the efficacy of vaccination services. The present study revealed that the rate of vaccine hesitancy (7.5%) and the rate of deciding not to vaccinate their children for reasons other than illness or allergy (8.0%) were found to be low. The analysis revealed a correlation between participants’ attitudes towards childhood vaccines and their gender, education level, and income level. Furthermore, parental attitudes were found to be influenced by both trust in childhood vaccines and adherence to them. It is imperative to implement training programmes that augment healthcare professionals’ knowledge about vaccines and confidence in their use, particularly among those with limited financial resources and educational attainment. Furthermore, the implementation of educational and communication programmes aimed at combating negative attitudes towards vaccination within the community is imperative. The enhancement of vaccine literacy among the public, notably healthcare professionals, the refinement of vaccine-related educational content, and the formulation of education and communication programmes for parents who do not vaccinate, are poised to assume pivotal roles in the management of vaccine hesitancy. The implementation of epidemiological studies focusing on the causes of vaccine refusal and hesitancy will facilitate a more comprehensive understanding of the reasons behind vaccine hesitancy, thereby contributing to the development of appropriate solutions.

## Supplementary Information

Supplementary Material 1.

## Data Availability

No datasets were generated or analysed during the current study.

## Abbreviations

PACV: Parental attitudes toward childhood vaccines
5C scale: psychological vaccination antecedents
WHO: World Health Organisation
SPSS: The Statistical Package for the Social Sciences
SD: Standard deviation
TRY: Turkish Lira
