# Supplementary material for: Vaccine hesitation: attitudes of Turkish health worker parents towards adult and childhood immunisation
Source: BMC Public Health. 2025 Aug 20;25:2857. doi: 10.1186/s12889-025-24100-5 (PMC12365999; doi:10.1186/s12889-025-24100-5)
Supplement: Supplementary file 1 — Supplementary Material 1. [file 12889_2025_24100_MOESM1_ESM.docx]

Vaccine Hesitancy: The Attitudes Of Healthcare Workers With Children Regarding Adult And Childhood Immunisation

You have been invited to participate in this study. Before accepting to take part in this study, you must understand the purpose of the study and make your decision freely after this information. Please read this information prepared specifically for you carefully. Ask for clear answers to your questions.

The purpose of the study is to determine the attitudes and hesitations of health workers towards their own adult vaccinations and childhood vaccination as parents. The information you provide will be used only for scientific purposes and no personal information, especially your identity, will be shared with third parties.

You are free to participate or not participate in the study. You can refuse to participate in the study at any time without being subject to any sanctions and without losing any of your rights, or you can withdraw from the study at any time.

You can reach the person whose name and phone number are given below during this research period.

Contact Persons: Dr. Emre ÇELİK Tel: +90 532 100 62 44

Dr Çağrı Emin ŞAHİN Tel: +90 505 282 33 01

Dr. Mehmet Sait DEĞER Tel: +90 532 674 50 12

Dr. Zeynep Meva ALTAŞ Tel: +90 535 735 62 35

1. Age:

2. Gender: a) Female b) Male

3. Education: a) Secondary School b) High School c) Bachelor's Degree d) Master's Degree e) Doctorate and above

4. Profession: a) Physician b) Midwife/Nurse/Health Officer c) Technician/Technician d) Other

5. Academic Title (if any):

6. Professional Experience: a) Less than 1 year b) 1-5 years c) 6-10 years d) 11-15 years e) Over 15 years

7. Your Income Level: a) Below 30 thousand TL b) 30 thousand TL-50 thousand TL c) 50 thousand-80 thousand d) Over 80 thousand

8. Child number:

9. Age of Youngest Child:

**Parental Attitudes About Childhood Vaccinations Scale**

1. Have you ever delayed your child’s vaccinations for reasons other than illness or allergies (except seasonal flu or swine flu (H1N1) vaccines)?

a) Yes   b) No  c) Don’t know

2. Have you ever decided not to vaccinate your child for reasons other than illness or allergies (except seasonal flu or swine flu (H1N1) vaccines)?

a) Yes  b) No  c) Don’t know

3. If you had another baby today, would you want him/her to receive all the recommended vaccines?

a) Yes   b) No  c) Don’t know

4. How confident are you that following the recommended vaccination schedule is a good idea for your child? Please answer on a scale of 0 to 10; where 0 means “Not Confident at All” and 10 means “Absolutely Confident.”

5. All things considered, how much do you trust your child’s doctor? Please answer on a scale of 0 to 10, where 0 means “Not Confident at All” and 10 means “Absolutely Confident.”

Rate each question from 1 to 5.

Strongly Agree 5

Agree 4

Not Confident 3

Disagree 2

Strongly Disagree 1

6. Children get more vaccines than they need.

7. I believe that most of the diseases that vaccines prevent are serious.

8. It is better for my child to get sick and develop immunity than to get vaccinated.

9. It is better for children to get fewer vaccines at a time.

10. I worry that my child could have serious side effects from any vaccine.

11. I worry that any childhood vaccines may not be safe.

12. I worry that a vaccine will not prevent a disease.

13. In general, I am hesitant about childhood vaccinations.

14. I trust the information I receive about vaccines.

15. I can openly discuss my concerns about vaccines with my child's doctor.

**Psychological Antecedents of Vaccination (5C) Scale**

Rate each question from 1 to 5.

Strongly agree 5

Agree 4

Not sure 3

Disagree 2

Strongly disagree 1

Confidence

1. I am confident that vaccines are safe

2. Vaccines are effective

3. I am confident that authorities will make decisions about vaccines in the best interest of society.

4. Since vaccine-preventable diseases are decreasing, there is no need to get vaccinated.

5. My immune system is quite strong, which protects me from diseases.

6. Since vaccine-preventable diseases are not very severe, there is no need for me to get vaccinated.

Constraint

7. Daily hustle and bustle may prevent me from getting vaccinated.

8. Getting vaccinated is a hassle for me.

9. I avoid getting vaccinated because going to the doctor makes me feel uncomfortable.

Calculation

10. When deciding to get vaccinated, I consider all the benefits and risks to make the best possible decision.

11. Every time I get vaccinated, I carefully consider whether the vaccine is beneficial for me.

12. Before getting vaccinated, it is important for me to have full information about the vaccine.

Collective responsibility

13. If everyone gets vaccinated, I don't need to get vaccinated.

14. I get vaccinated because I can also protect people with weaker immune systems by getting vaccinated.

15. Getting vaccinated is a social action to prevent the spread of diseases.
